# Epidural Analgesia and Its Impact on the Duration of the Second Stage of Labor, Vacuum Extraction Delivery, and Neonatal Apgar Scores: A Retrospective Cohort Study

**DOI:** 10.3390/medicina62081535

**Published:** 2026-08-10

**Authors:** Wing Lam Tsui, Dah-Ching Ding

**Affiliations:** 1 Department of Obstetrics and Gynecology, Hualien Tzu Chi Hospital, Buddhist Tzu Chi Medical Foundation, Tzu Chi University, Hualien 970374, Taiwan; karenwinglam0505@gmail.com; 2 Institute of Medical Sciences, Tzu Chi University, Hualien 970374, Taiwan

**Keywords:** epidural analgesia, vacuum extraction delivery, second stage of labor, Apgar score, intrapartum outcomes

## Abstract

*Background and Objectives*: Epidural analgesia is widely used for intrapartum pain relief; nonetheless, its associations with labor progression and neonatal outcomes remain incompletely characterized in diverse obstetric populations. This retrospective cohort study aimed to investigate the association between labor epidural analgesia and obstetric outcomes, with a focus on the duration of the second stage of labor and the vacuum extraction delivery (VED) rate. *Materials and Methods*: Maternal and neonatal outcomes were compared between 173 women without epidural analgesia and 386 women with epidural analgesia at a single tertiary center. Independent predictors of VED, duration of the second stage of labor, and 1-min Apgar score ≤ 6 were identified by conducting multivariate logistic and linear regression analyses. *Results*: After adjustment for maternal age, body mass index, gestational age, parity, labor induction, and comorbidities, epidural analgesia was identified as the strongest independent predictor across all three outcomes. Notably, epidural analgesia was associated with nearly 6.7-fold higher odds of VED (adjusted odds ratio [aOR]: 6.74, 95% confidence interval [CI]: 3.55–12.82), approximately 49 additional min for second-stage labor (β = 48.63, 95% CI: 32.74–64.52), and approximately 5-fold higher odds of low 1-min Apgar score (aOR: 5.14, 95% CI: 1.11–23.87). Higher parity and labor induction were independently associated with a shorter duration of the second stage of labor and with lower odds of instrumental delivery. These associations remained robust after propensity score matching for parity and other baseline covariates, as well as in parity-stratified subgroup analysis (all *p* < 0.05). *Conclusions*: These findings highlight the multifaceted obstetric impact of epidural analgesia and underscore the importance of individualized intrapartum monitoring and informed patient counseling.

## 1. Introduction

Labor pain is a complex physiological and psychological experience that can substantially affect maternal well-being during childbirth [1]. Labor epidural analgesia (LEA), commonly referred to as “painless labor,” is considered the most effective pain relief method during labor and has been increasingly used worldwide [2]. LEA not only allows patients to remain conscious and actively participate in the birthing process but also contributes considerably to improvements in maternal satisfaction and overall childbirth experience [3].

Despite the established analgesic benefits of LEA, its influence on labor outcomes remains a topic of ongoing debate. Epidural analgesia has been suggested by previous studies to alter the labor course by reducing mothers’ bearing-down efforts and affecting their uterine contractility, potentially resulting in a prolonged second stage of labor [4,5]. Furthermore, concerns about an increased likelihood of operative vaginal delivery—particularly, vacuum extraction or forceps-assisted birth—among women receiving epidural analgesia have been raised [5]. Nonetheless, these associations have varied across studies, and findings remain inconsistent owing to differences in study populations, obstetric practices, and analgesic techniques.

Accordingly, the present retrospective cohort study aimed to investigate the association between LEA and obstetric outcomes, with a focus on the duration of the second stage of labor and the vacuum extraction delivery (VED) rate.

## 2. Methods

### 2.1. Patient Selection

This single-institution retrospective cohort study was conducted at Hualien Tzu Chi Hospital. This study was conducted in accordance with the principles embodied in the Declaration of Helsinki and with relevant ethical standards and guidelines and was approved by the Research Ethics Committee of Hualien Tzu Chi Hospital (approval code: IRB114-141-B; approval date: 29 July 2025). The requirement for informed consent was waived by the Research Ethics Committee owing to the retrospective nature of this study. Patients’ data were retrieved from the hospital’s electronic medical records.

### 2.2. Inclusion and Exclusion Criteria

This study included women who delivered at Hualien Tzu Chi Hospital from 2022 to 2023. The exclusion criteria were as follows: (i) preterm birth (gestational age [GA] < 37 weeks), (ii) vaginal birth after a previous cesarean section (VBAC, given the distinct intrapartum management protocols and uterine rupture risk associated with VBAC, which could confound the assessment of second-stage duration and mode of delivery), (iii) missing data, and (iv) immediate neonatal death after birth or termination cases.

### 2.3. Patient Characteristics

The basic characteristics of the recruited participants, including age, body mass index (BMI), parity, GA, labor induction, delivery method, blood loss, birth weight, duration of the first and second stage of labor, maternal complications (diabetes mellitus [DM], hypertension, and preeclampsia), and 1-min and 5-min Apgar scores, were recorded.

### 2.4. Outcome Measures

The primary outcome was the duration of the second stage of labor. The duration of the second stage of labor was defined as the interval from complete cervical dilation (10 cm) to delivery of the neonate and was analyzed as a continuous variable (minutes) rather than as a dichotomized diagnosis of ‘prolonged labor.’ This approach was chosen to preserve statistical power and to allow quantification of the magnitude of association between epidural analgesia and second-stage duration, rather than relying on threshold-based clinical definitions of labor arrest or prolongation (ACOG criteria [6]), which were not applied as a categorical outcome in this study. The secondary outcomes were the delivery method with VED and the neonatal outcomes (Apgar score ≤ 6).

To further address potential confounding by parity, 1:1 propensity score matching (PSM) was performed incorporating parity status together with maternal age, BMI, gestational age, labor induction, and comorbidities, yielding a matched cohort of 167 women per group (standardized mean differences < 0.12 for all covariates, Table S1). Outcomes were re-estimated in the matched cohort, and a parity-stratified subgroup analysis (nulliparous vs. multiparous) was additionally performed.

### 2.5. Painless Labor Procedure

Painless labor is indicated during active vaginal labor upon maternal request to relieve severe pain, exhaustion, or anxiety, or medically when intensive contractions are induced or stress reduction is needed for conditions like preeclampsia. In our hospital, anesthesiologists performed the painless labor procedure, also referred to as “epidural anesthesia”. The catheter is usually placed before active labor begins, and we usually start infusion at cervical os open to 3–4 cm. An intravenous line was started, and vital signs were monitored. Preloading was applied with Ringer’s lactate solution (15 mL/kg body weight). Patients were instructed to sit or curl onto their side. The L3–4 or L4–5 intervertebral space was identified while exercising aseptic precautions, and 2% lignocaine hydrochloride (3 mL) was injected locally. An epidural catheter was inserted into the epidural space using an 18-gauge Tuohy needle. Subsequently, a 6-mL bolus of 0.1% bupivacaine with fentanyl (2 μg/mL) was administered, followed by continuous infusion at 4 mL/h using the same regimen.

### 2.6. Statistical Analysis

Continuous variables are expressed as mean ± standard deviation. Dichotomous variables were examined using Fisher’s exact test or chi-squared test, whereas continuous variables were analyzed using independent Student’s *t*-test to determine statistical significance. Multivariate logistic regression analysis adjusted for study covariates was conducted on the entire cohort to estimate the adjusted odds ratio (aOR) for painless labor and assess its association with neonatal outcomes or VED. Multivariate linear regression analysis was conducted for the duration of the second stage of labor. All statistical analyses were performed using SPSS version 25 (IBM Corp., Armonk, NY, USA), with statistical significance set at *p* < 0.05.

## 3. Results

### 3.1. Study Flow

**Figure 1:**
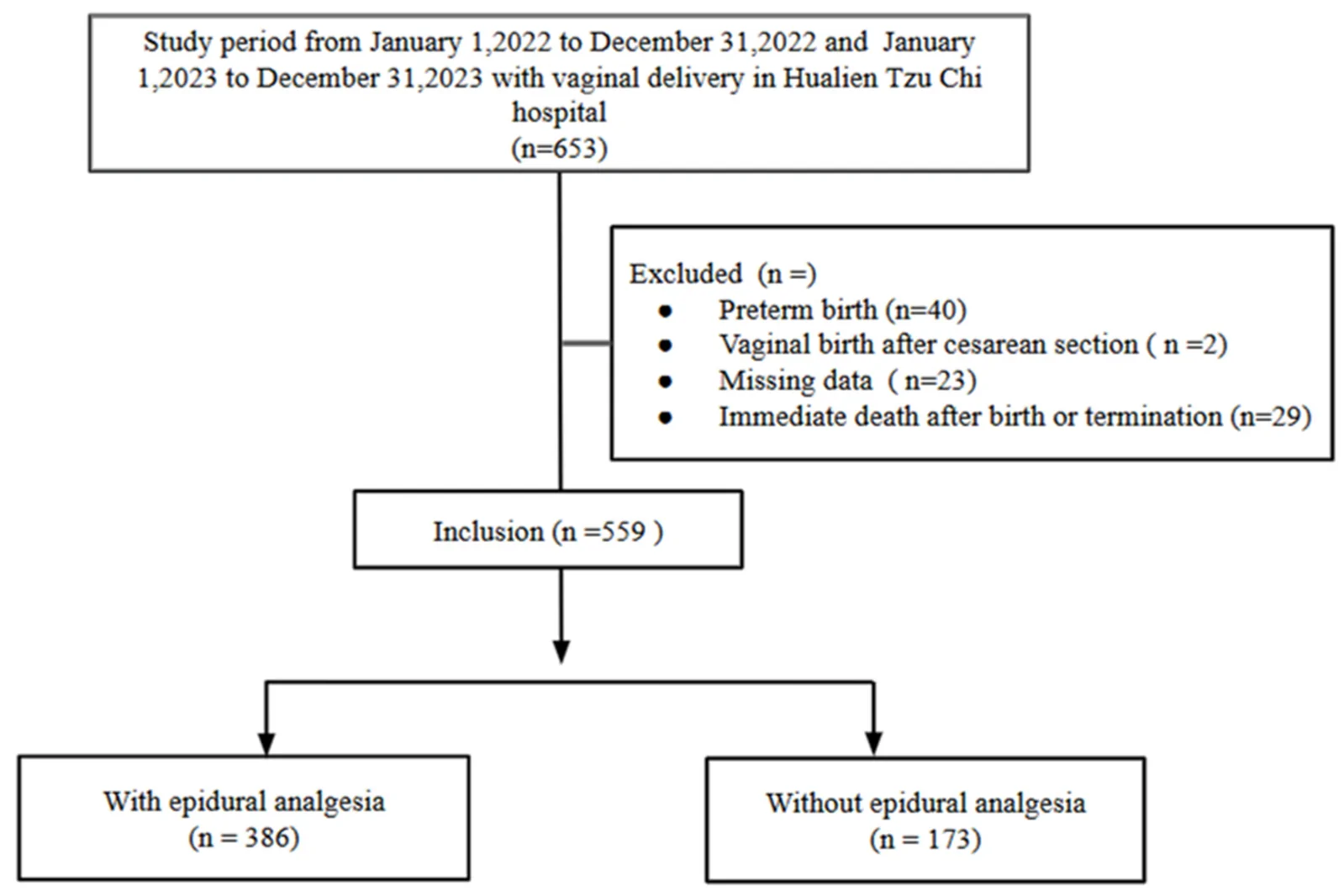
Study flow chart.

Of 653 women who delivered vaginally at Hualien Tzu Chi Hospital between January 2022 and December 2023, 559 met the inclusion criteria after excluding preterm births, VBAC, missing data, and immediate neonatal death or termination (Figure 1). The final cohort comprised 386 women (69.1%) with epidural analgesia and 173 (30.9%) without.

### 3.2. Basic Characteristics

Baseline maternal and obstetric characteristics are summarized in Table 1. Labor induction was common (60.3%), and one-quarter of participants (25.0%) delivered via VED. Full descriptive statistics are presented in Table 1.

### 3.3. Comparison of Maternal and Neonatal Outcomes Between Those With or Without Epidural Analgesia

Women who received epidural analgesia had lower parity and were more frequently induced than those without (Table 2). Compared with the no-epidural group, the epidural group had a markedly higher VED rate, a longer second stage of labor, and a modestly lower 1-min Apgar score, including a higher proportion of scores ≤ 6 (all *p* < 0.05; Table 2). Maternal age, BMI, GA, and comorbidity prevalence did not differ significantly between groups.

### 3.4. Factors Associated with the Incidence of VED

On multivariate logistic regression, epidural analgesia was the strongest independent predictor of VED, conferring nearly 6.7-fold higher odds of instrumental delivery (Table 3). Higher parity and labor induction were independently associated with modestly lower odds of VED, whereas maternal age, BMI, GA, and comorbidities were not significant predictors.

### 3.5. Factors Associated with the Duration of the Second Stage of Labor

Epidural analgesia was independently associated with a longer second stage of labor, adding approximately 49 min compared with no epidural analgesia (Table 4). In contrast, higher parity and labor induction were each independently associated with a shorter second stage. BMI, maternal age, GA, and comorbidities showed no significant association.

### 3.6. Factors Associated with Poorer Neonatal Outcomes

Epidural analgesia was the only variable independently associated with a 1-min Apgar score ≤ 6, conferring approximately 5-fold higher odds; however, the wide confidence interval warrants cautious interpretation (Table 5). No other covariate reached statistical significance.

### 3.7. Sensitivity Analysis: Propensity Score Matching and Parity-Stratified Subgroup Analysis

After 1:1 PSM balancing parity and other baseline covariates (Table S1), epidural analgesia remained independently associated with a higher VED rate (OR: 6.87, 95% CI: 3.52–13.41, *p* < 0.001), a longer second stage of labor (β = 59.64 min, 95% CI: 44.32–74.96, *p* < 0.001), and higher odds of 1-min Apgar score ≤ 6 (OR: 4.70, 95% CI: 1.00–22.09, *p* = 0.050) (Table S2). Parity-stratified analysis confirmed that these associations persisted in both nulliparous and multiparous women, with a somewhat greater magnitude of association with VED among multiparous women (OR: 9.56 vs. 3.83) (Table S3).

## 4. Discussion

This retrospective cohort study compared maternal and neonatal outcomes between 173 women without epidural analgesia and 386 women with epidural analgesia. Across all three outcome domains, epidural analgesia was identified as the strongest independent predictor after multivariate adjustment. Women who received epidural analgesia showed nearly 6.7-fold higher odds of VED, had approximately 49 additional min for second-stage labor, and exhibited approximately 5-fold higher odds of 1-min Apgar score ≤ 6. Higher parity and labor induction were independently associated with more favorable labor outcomes, whereas comorbidities did not significantly influence any endpoint. These associations remained robust after propensity score matching for parity and other baseline covariates, as well as in parity-stratified subgroup analysis.

Consistent with our findings, previous studies have identified epidural analgesia as a significant independent predictor of prolonged second-stage labor. Pham et al. (2022) found epidural analgesia to be among the strongest determinants of prolonged second-stage labor in a national French cohort, particularly among nulliparous women, reflecting its systemic effect on expulsive efforts and pelvic floor relaxation [7]. Zheng et al. (2020) similarly reported that continuing epidural analgesia into the second stage was associated with a measurably longer pushing duration, consistent with the motor blockade effect attenuating voluntary expulsive force [8]. Miyamoto et al. (2024) further linked epidural-associated second-stage prolongation to downstream complications such as postpartum urinary retention, underscoring its clinical relevance beyond delivery outcomes alone [9]. Cohen et al. (2025) reported worse composite outcomes among nulliparous women with epidural analgesia whose second stage exceeded four hours, reinforcing the clinical threshold relevance of this prolongation [4], while Newnham et al. (2021) reported a comparable magnitude of second-stage prolongation among nulliparous women with epidural analgesia [10].

Our observation of a substantially increased VED rate with epidural analgesia aligns with a broad body of evidence linking neuraxial analgesia to instrumental delivery. Srebnik et al. (2020) attributed this association to reduced maternal urge to push and impaired pelvic floor sensation among nulliparous women [11]. Lipschuetz et al. (2020) found the effect to be independent of cervical dilation at catheter insertion, suggesting an effect intrinsic to the analgesia itself rather than timing-dependent [12]. Newnham et al. (2021) and Erickson et al. (2024) similarly reported higher instrumental delivery rates with epidural use, the latter highlighting an interaction with passive descent and pushing management strategies [10,13]. Turner et al. (2020), however, noted that this association was not accompanied by increased maternal or neonatal morbidity, suggesting the excess instrumental delivery risk does not necessarily translate into worse outcomes [14].

Our finding that epidural analgesia was the sole independent predictor of a low 1-min Apgar score requires cautious interpretation, as the literature on this association is heterogeneous. Turner et al. (2020) found no association between epidural use and neonatal morbidity even with second-stage prolongation, suggesting adverse neonatal outcomes may be mediated by labor duration or delivery mode rather than analgesia itself [14]. This is supported by Nelson et al. (2020), who linked prolonged second-stage labor per se to increased low Apgar scores and neonatal acidemia [15], and by Zheng et al. (2020), who found that epidural continuation did not independently worsen Apgar scores after adjustment for delivery mode [8]. Di Mascio et al. (2020) similarly found no Apgar score difference by pushing timing among women with neuraxial analgesia [16]. Collectively, these findings suggest that the neonatal signal observed in our cohort may reflect a composite effect of prolonged second-stage labor and higher instrumental delivery rates rather than a direct pharmacological effect of epidural agents on neonatal adaptation.

Our finding that higher parity was protective against second-stage prolongation and instrumental delivery is consistent with established evidence that multiparous women experience more efficient labor progression due to prior cervical remodeling, greater pelvic floor compliance, and more effective expulsive efforts. Pham et al. (2022) and Nelson et al. (2020) both identified nulliparity as a major independent predictor of second-stage prolongation and operative delivery, regardless of epidural use [7,15], and Newnham et al. (2021) similarly found multiparity to be one of the most robust protective factors for labor progression [10].

The counterintuitive finding that labor induction was independently associated with reduced VED rate and shorter second-stage duration may reflect the structured clinical management and closer intrapartum surveillance typically accompanying induction protocols. Lipschuetz et al. (2020) suggested that cervical preparation and controlled labor progression in induced patients yield more predictable second-stage trajectories than spontaneous labor complicated by prolonged epidural exposure [12]. Gu et al. (2020) similarly found that induced labor cohorts demonstrated distinct second-stage characteristics compared with spontaneous labor [17], and Turner et al. (2020) observed that structured intrapartum management accompanying induction was not associated with increased instrumental delivery when appropriate monitoring was in place, indirectly supporting a protective role for this coordinated care environment [14].

### Strengths and Limitations

This study benefits from a relatively large sample size with clearly defined comparison groups, allowing for multivariate regression analyses adjusted for key confounders such as maternal age, BMI, GA, parity, labor induction, and comorbidities. The use of both logistic and linear regression models enabled a comprehensive evaluation of several clinically relevant outcomes, including VED, duration of the second stage of labor, and neonatal Apgar score, providing a multidimensional picture of the effects of epidural analgesia on obstetric outcomes. The inclusion of comorbidity variables such as preeclampsia, hypertension, and DM further strengthens the validity of adjusted estimates by minimizing residual confounding from underlying maternal conditions.

This study has some limitations. First, its single-center retrospective design limits generalizability and may introduce selection bias, as women choosing epidural analgesia may differ systematically from those who did not in unmeasured ways. Second, the small number of low-Apgar events (*n* = 21) produced wide confidence intervals for that outcome, reducing precision. Third, unmeasured variables such as fetal weight, anesthesiologist experience, oxytocin dosing, and pain tolerance were not captured. Additionally, women attempting VBAC were excluded and findings should not be extrapolated to that population; second-stage duration was analyzed continuously rather than by standardized prolonged-labor criteria; and specific indications for VED were not available, though these may differ by epidural status and independently affect second-stage duration. Although our institutional protocol standardly places the epidural catheter early in active labor (cervical dilation 3–4 cm), catheter timing was not recorded case-by-case, so we cannot fully exclude late placement for anticipated instrumental delivery, which could introduce reverse-causation bias. Finally, as an observational study, causal inference cannot be established, and prospective randomized studies are needed to confirm these associations.

## 5. Conclusions

This study revealed that epidural analgesia was independently associated with, rather than shown to cause, a significantly prolonged second stage of labor, higher VED rate, and increased odds of a low 1-min Apgar score, and these associations remained robust after propensity score matching on parity and other baseline covariates, as well as in parity-stratified subgroup analysis. However, because the clinical indication for VED could not be ascertained, these associations may partly reflect confounding by indication (e.g., fetal distress prompting both VED and a low Apgar score independent of epidural analgesia) rather than a direct causal pathway. Higher parity and labor induction were independently associated with a shorter second stage of labor and lower odds of instrumental delivery, respectively, but did not account for the epidural-associated risk. These findings support close intrapartum monitoring and individualized birth planning for women receiving epidural analgesia, particularly nulliparous women and those undergoing labor induction, but should not be used to imply that epidural analgesia itself causes operative delivery or neonatal depression. Future prospective studies with indication-level data on VED are needed to clarify whether epidural analgesia is a cause of, or a marker for, more complicated labor courses.

## Figures and Tables

**Table 1:** Baseline characteristics of the study population (*n* = 559).

Variable	Total (*n* = 559)
Maternal age, mean ± SD	31.35 ± 13.43
BMI, mean ± SD	26.94 ± 4.76
GA week, mean ± SD	38.69 ± 0.76
Parity, mean ± SD	1.18 ± 1.14
Induction, *n* (%)	337 (60.3%)
Prostaglandin E2 tablets	167 (29.9%)
Dinoprostone delivery system	169 (30.2%)
Oxytocin	1 (0.2%)
Comorbidity, *n* (%)	
Preeclampsia	4 (0.7%)
DM	52 (9.3%)
Hypertension	16 (2.9%)
Delivery method, *n* (%)	
Normal spontaneous delivery	419 (75.0%)
VED	140 (25.0%)
Uterotonic agent, *n* (%)	
Oxytocin	85 (15.2%)
Duratocin	474 (84.8%)
Epidural analgesia, *n* (%)	
No	173 (30.9%)
Yes	386 (69.1%)
Birth height (cm), mean ± SD	48.68 ± 1.79
Birth weight (g), mean ± SD	3019.99 ± 324.44
Blood loss (mL), mean ± SD	217.05 ± 242.58
1-min Apgar score, median (IQR)	8 (1)
3-min Apgar score, median (IQR)	9 (0)
First stage labor (min), mean ± SD	815.52 ± 623.46
Second stage labor (min), mean ± SD	69.48 ± 90.84

BMI, body mass index; GA, gestational age; SD, standard deviation; IQR, interquartile range; DM, diabetes mellitus; and VED, vacuum extraction delivery.

**Table 2:** Comparison of maternal and neonatal outcomes between women with and without epidural analgesia.

Variable	No Epidural Analgesia (*n* = 173)	Epidural Analgesia (*n* = 386)	*p*-Value
	Mean ± SD or *n* (%)	Mean ± SD or *n* (%)	
Maternal age (years), mean ± SD	32.78 ± 22.89	30.70 ± 5.10	0.240
BMI (kg/m^2^), mean ± SD	27.47 ± 5.09	26.70 ± 4.58	0.078
GA week, mean ± SD	38.67 ± 0.79	38.70 ± 0.75	0.677
Parity, mean ± SD	1.73 ± 1.41	0.93 ± 0.89	<0.001
Induction, *n* (%)	71 (41.0%)	266 (68.9%)	<0.001
Comorbidity, *n* (%)			
Preeclampsia	0 (0%)	4 (1.0%)	0.317
DM	17 (9.8%)	35 (9.1%)	0.775
Hypertension	4 (2.3%)	12 (3.1%)	0.786
Delivery method, *n* (%)			<0.001
Normal spontaneous delivery	160 (92.5%)	259 (67.1%)	
VED	13 (7.5%)	127 (32.9%)	
Second stage labor (min), mean ± SD	26.77 ± 52.62	88.63 ± 97.65	<0.001
1-min Apgar score, mean ± SD	8.27 ± 0.65	8.03 ± 0.94	0.001
1-min Apgar score, *n* (%)			
Score > 6	171 (98.8%)	367 (95.1%)	0.030
Score ≤ 6	2 (1.2%)	19 (4.9%)	

BMI, body mass index; GA, gestational age; SD, standard deviation; DM, diabetes mellitus; and VED, vacuum extraction delivery.

**Table 3:** Multivariate logistic regression analysis for VED.

Variable	aOR (95% CI)	*p*-Value
BMI	1.02 (0.97–1.06)	0.480
Maternal age	1.00 (0.99–1.02)	0.797
GA week	1.15 (0.88–1.52)	0.311
Parity	0.75 (0.57–0.99)	0.045
Induction (ref: no)	0.54 (0.35–0.84)	0.007
Comorbidity (ref: no)		
Preeclampsia	1.14 (0.07–18.81)	0.926
Hypertension	0.61 (0.12–3.19)	0.554
DM	1.48 (0.74–2.95)	0.266
Epidural analgesia (ref: no)		
Yes	6.74 (3.55–12.82)	<0.001

aOR, adjusted odds ratio; CI, confidence interval; BMI, body mass index; GA, gestational age; and DM, diabetes mellitus.

**Table 4:** Multivariate linear regression analysis for the duration of the second stage of labor (minutes).

Variable	Adjusted β (95% CI)	*p*-Value
BMI	0.47 (−0.99 to 1.94)	0.527
Maternal age	0.08 (−0.42 to 0.59)	0.747
GA week	4.21 (−5.01 to 13.42)	0.371
Parity	−38.77 (−47.90 to −29.65)	<0.001
Induction (ref: no)	−15.55 (−30.25 to −0.84)	0.038
Comorbidity (ref: no)		
Hypertension	−15.75 (−63.83 to 32.33)	0.520
DM	−5.60 (−29.12 to 17.91)	0.640
Preeclampsia	−25.73 (−118.48 to 67.01)	0.586
Epidural analgesia (ref: no)		
Yes	48.63 (32.74 to 64.52)	<0.001

CI, confidence interval; BMI, body mass index; GA, gestational age; and DM, diabetes mellitus.

**Table 5:** Multivariate logistic regression analysis for 1-min Apgar score ≤ 6.

Variable	aOR (95% CI)	*p*-Value
BMI	1.06 (0.98–1.16)	0.163
Maternal age	1.01 (0.98–1.04)	0.747
GA week	1.11 (0.61–2.04)	0.734
Parity	0.82 (0.44–1.51)	0.521
Induction (ref: no)	0.58 (0.23–1.47)	0.252
Comorbidity (ref: no)		
Preeclampsia	–	–
Hypertension	1.80 (0.18–18.34)	0.620
DM	1.53 (0.40–5.84)	0.532
Epidural analgesia (ref: no)		
Yes	5.14 (1.11–23.87)	0.037

aOR, adjusted odds ratio; CI, confidence interval; BMI, body mass index; GA, gestational age; and DM, diabetes mellitus.

## Data Availability

Sharing the data of this article is not permissible because of data protection regulations.

## Supplementary Materials

The following supporting information can be downloaded at: https://www.mdpi.com/article/10.3390/medicina62081535/s1, Table S1: Baseline characteristics after 1:1 propensity score matching between women with and without epidural analgesia; Table S2: Association between epidural analgesia and maternal/neonatal outcomes after propensity score matching, adjusted for parity status; Table S3: Subgroup analysis of the association between epidural analgesia and outcomes, stratified by parity status. Table S4: Nulliparous and multiparous descriptive characteristics.

## Abbreviations

The following abbreviations are used in this manuscript:

aOR	Adjusted odds ratio
BMI	Body mass index
CI	Confidence interval
DM	Diabetes mellitus
GA	Gestational age
LEA	Labor epidural analgesia
VED	Vacuum extraction delivery
