# Supplementary material for: Epidural Analgesia and Its Impact on the Duration of the Second Stage of Labor, Vacuum Extraction Delivery, and Neonatal Apgar Scores: A Retrospective Cohort Study
Source: Medicina (Kaunas). 2026 Aug 10;62(8):1535. doi: 10.3390/medicina62081535 (PMC13515525; doi:10.3390/medicina62081535)
Supplement: Supplementary file 1 [file medicina-62-01535-s001.zip › medicina-4408100-supplementary.pdf]

Table S1. Baseline characteristics after 1:1 propensity score matching between women with and without epidural analgesia.

	**No epidural analgesia** (n=167)	**Epidural analgesia** (n=167)	smd
Maternal Age, Mean±SD	31.02±5.97	30.97±5.22	0.009
BMI, Mean±SD	27.33±4.95	26.76±4.72	0.118
GA week, Mean ±SD	38.67±0.8	38.66±0.75	0.013
Parity status, n (%)			
Nulliparous	35(20.96)	31(18.56)	0.060
Multiparous	132(79.04)	136(81.44)	0.060
Induction, n (%)	71(42.51)	80(47.90)	0.108
Disease, n (%)			
Preeclampsia	0(0.00)	0(0.00)	0.000
DM	16(9.58)	12(7.19)	0.086
Hypertension	4(2.40)	2(1.20)	0.090

Smd: Standardized Mean Difference. BMI: body mass index, GA: gestational age, DM: diabetes mellitus, SD: standard deviation.

Table S2. Association between epidural analgesia and maternal/neonatal outcomes after propensity score matching, adjusted for parity status.

	Estimate (95% CI)	P
VED	6.87 (3.52, 13.41)	<0.001
1st min APGAR<=6	4.70 (1.00, 22.09)	0.050
2nd stage labor	59.64 (44.32, 74.96)	<0.001

ORs were estimated using logistic regression, and β coefficients were estimated using linear regression.

VED: vacuum extraction delivery. CI: confidence interval.

Table S3. Subgroup analysis of the association between epidural analgesia and outcomes, stratified by parity status.

	Estimate (95% CI)	P
VED		
Multiparous	9.56 (4.32, 21.16)	<0.001
Nulliparous	3.83 (1.32, 11.15)	0.01
1st min APGAR<=6		
Multiparous	3.99 (0.82, 19.38)	0.087
Nulliparous	-	
2nd stage labor		
Multiparous	61.33 (46.85, 75.81)	<0.001
Nulliparous	52.64 (7.39, 97.90)	0.020

Estimate represents adjusted odds ratios for binary outcomes and adjusted β coefficients for continuous outcomes. VED: vacuum extraction delivery.

Table S4: Nulliparous and multiparous descriptive characteristics.

	**No epidural analgesia** (*n* = 173)	**Epidural analgesia** (*n* = 386)	p
Parity status, n (%)			0.0007
Nulliparous	35(20.83)	133(34.46)	
Multiparous	138(79.77)	253(65.54)	
